# Light Modulation for Bioactive Pigment Production in *Synechocystis salina*

**DOI:** 10.3390/bioengineering9070331

**Published:** 2022-07-21

**Authors:** Joana Assunção, Fernando Pagels, Tânia Tavares, F. Xavier Malcata, A. Catarina Guedes

**Affiliations:** 1 CIIMAR /CIMAR-LA—Interdisciplinary Centre of Marine and Environmental Research, University of Porto, Novo Edifício do Terminal de Cruzeiros do Porto de Leixões, Av. General Norton de Matos, s/n, 4450-208 Matosinhos, Portugal; joanaleonardoassuncao@gmail.com (J.A.); fernandopagels@gmail.com (F.P.); acatarinaguedes@gmail.com (A.C.G.); 2 LEPABE—Laboratory for Process Engineering, Environment, Biotechnology and Energy, Faculty of Engineering, University of Porto, Rua Dr. Roberto Frias, s/n, 4200-465 Porto, Portugal; tsgtavares@gmail.com; 3 FCUP—Faculty of Sciences, University of Porto, Rua do Campo Alegre, s/n, 4169-007 Porto, Portugal; 4 ALiCE—Associate Laboratory in Chemical Engineering, Faculty of Engineering, University of Porto, Rua Dr. Roberto Frias, 4200-465 Porto, Portugal; 5 FEUP—Department of Chemical Engineering, Faculty of Engineering, University of Porto, Rua Dr. Roberto Frias, s/n, 4200-465 Porto, Portugal

**Keywords:** light modulation, pigment, cyanobacterium, acclimatization, optimization

## Abstract

Cyanobacteria are microorganisms that are well-adapted to sudden changes in their environment, namely to light conditions. This has allowed them to develop mechanisms for photoprotection, which encompass alteration in pigment composition. Therefore, light modulation appears to be a suitable strategy to enhance the synthesis of specific pigments (e.g., phycocyanin) with commercial interest, in addition to conveying a more fundamental perspective on the mechanisms of acclimatization of cyanobacterium species. In this study, *Synechocystis salina* was accordingly cultivated in two light phase stages: (i) white LED, and (ii) shift to distinct light treatments, including white, green, and red LEDs. The type of LED lighting was combined with two intensities (50 and 150 µmol_photons_·m^−2^·s^−1^). The effects on biomass production, photosynthetic efficiency, chlorophyll *a* (chl *a*) content, total carotenoids (and profile thereof), and phycobiliproteins (including phycocyanin, allophycocyanin, and phycoerythrin) were assessed. White light (under high intensity) led to higher biomass production, growth, and productivity; this is consistent with higher photosynthetic efficiency. However, chl *a* underwent a deeper impact under green light (high intensity); total carotenoids were influenced by white light (high intensity); whilst red treatment had a higher effect upon total and individual phycobiliproteins. Enhanced PC productivities were found under modulation with red light (low intensities), and could be achieved 7 days earlier than in white LED (over 22 days); this finding is quite interesting from a sustainability and economic point of view. Light modulation accordingly appears to be a useful tool for supplementary studies pertaining to optimization of pigment production with biotechnological interest.

## 1. Introduction

Cyanobacteria are photosynthetic microorganisms that are widely adapted to environmental conditions across the globe. They can be divided into “sun” and “shade” species, depending on the light intensity required for growth thereof [1,2]. Said organisms are able to modify their photosynthetic apparatus to efficiently harvest light under fluctuating light conditions, and rapidly change from sub- to oversaturating light intensities. Therefore, cyanobacterial pigments have evolved to play an important role in the acclimation process, in particular to light. As major constituents of light-harvesting systems (LHS) and photosynthetic apparatuses—photosystem I (PSI), photosystem II (PSII), and phycobilisome, pigments are responsible to harvest light, and selectively absorbing at specific light wavelengths within the photosynthetic active radiation (PAR) range—a segment of the electromagnetic radiation spectrum, within 380–750 nm [3,4]. For this reason, cyanobacterial pigments, including chlorophyll *a* (chl *a*, λ_max_ = 430 and 660), carotenoids (λ = 400–500 nm), and phycobiliproteins (PBPs), such as phycocyanin (PC, λ_max_ = 620 nm), allophycocyanin (APC, λ_max_ = 650 nm), and phycoerythrin (PE λ_max_= 560 nm) are highly organized into the photosynthetic apparatus [4,5]. In addition to functioning as light receptors and funneling energy to the photoreaction centers, (i.e., chl *a* and PBPs), they also play an important role in cyanobacterial adaptation to external factors (e.g., high light intensities and nutrient limitation). Therefore, light promotes rearrangement of composition and content of cyanobacterial pigments, and several mechanisms of photoacclimation (and photoprotection) can actually be triggered [6]. When adjustment of the spectrum of nearly complementary to available light—the so-called complementary chromatic adaptation (CCA)—is activated, the ratio of PC and PE composing the phycobilisome (i.e., photosynthetic structure that harbors PBPs) is changed [6,7]. Cyanobacteria possess four types of chromatic acclimation in response to red/green light, which differs between species and is highly dependent upon pigment composition. Type I acclimation does not change PC or PE levels in response to light; in Type II, a change occurs in PE levels by both green and red light, whilst PC remains constant; in Type III, the PC levels are positively impacted by red light, whilst PE is positively favored by green light and vice-versa; and in the Type IV acclimation process, PBP content is regulated by blue and green light [8]. 

On the other hand, when light stress by high light quanta induces accumulation of reactive oxygen species (ROS) in cyanobacterial cells, some of the first active cell defenses are activated, while mechanisms of carotenoid synthesis are provided for photoprotection [9]. Therefore, cyanobacteria are highly adaptable to quality (i.e., specific wavelength), quantity (i.e., intensity), and/or duration (i.e., photoperiod) of light under different environments [10]. This means that light fluctuations can physiologically influence synthesis of important bioactive pigments, including chl *a*, carotenoids, and PBPs, thus playing a vital role in cyanobacterial growth, and pigment accumulation in culture [11]. 

From a biotechnological perspective, marine natural pigments (e.g., from cyanobacteria) have been found to possess outstanding natural properties (e.g., antioxidant, antitumor, anti-inflammatory, antiviral). They are used as ingredients in formulation of drugs, food (e.g., natural colorants, antioxidants), feed, cosmetics (e.g., anti-ageing), and therapeutical diagnostic (e.g., fluorescent dyes), as well as nutraceutical formulae for human and animal welfare (e.g., aquaculture and pets) [12,13,14]. The global market of natural pigments is currently undergoing considerable expansion. For instance, the total market of PBPs was estimated in 2018 at ca. USD 60 million, and is expected to double by 2028, with a value of USD 124 million [15]. 

In the upcoming future, enhanced biotechnological production of cyanobacterial bioactive pigments is expected to resort to innovative and cost-effective industrial technologies—such as light emitting diodes (LED). In this regard, a better understanding of how abiotic factors (e.g., light, temperature, nutrients) influence biomass and pigment accumulation, particularly in photoautotrophic cultivation systems, becomes crucial. Therefore, light modulation emerges as a relevant strategy to improve both biomass and pigment production in cyanobacteria.

The use of new light technologies, such as LED, provides a sustainable alternative to conventional lamps (e.g., halogen, fluorescent, high pressure, sodium) [16,17]. Despite the higher cost of installation (but with declining prices throughout the years), LEDs have a long life and entail low energy usage compared to conventional lighting. This could compensate the initial investment to some extent, and provide a more environment-friendly solution [18]. Several studies employing LED technology have shown their efficiency towards cyanobacterial culture and pigment modulation (e.g., *Arthrospira* spp.) [17,19,20]. The intensity and light wavelength can be tuned toward production of specific bioactive pigments, in terms of quantity and quality, along with biomass production [17]. However, optimal conditions are always dependent on the species chosen for that purpose. For instance, both red and/or green light favor production of PC/PE, as well as biomass production, depending on the species and strain at stake [12,17]. Furthermore, white light under “optimum” intensity—lower than saturation and photo-inhibition points—has been reported to favor the accumulation of biomass. It is important to reach a compromise between biomass production and level of pigments of interest. Therefore, use of two-phase cultivation is a commonly explored technique—at a first stage to maximize biomass (preferentially under higher growth rates), and at a second phase to target compounds enhanced by stress conditions (i.e., high light intensities, different light spectra) [21]. 

The marine cyanobacterium *Synechocystis salina* LEGE 01655 (*S. salina*) was previously described as having a potential biotechnological value [22,23,24]. Few studies on *Synechoscystis* species have, however, focused on light preferences, especially *Synechocystis* sp. strain PCC 6803 [25,26]—a strain mainly composed of APC and PC [27]. *S. salina* LEGE 01655 was found to have PE as additional pigment in its phycobilisome [22], but a gap remains regarding how light influences its metabolic responses. The process of light modulation is indeed highly dependent on the pigment composition of each organism, with a strain-specific response [25].

This work was accordingly aimed at assessing how light modulation under different wavelengths (white, green, and red) and intensities (high and dimmed light), in the two-phase cultivation of *S. salina*, can improve production of different pigments with added value (i.e., chl *a*, carotenoids, PBPs, and specifically PC—which were reported to high levels in this cyanobacterium [22]); and contributing to understand how this specie implements its chromatic acclimation. 

## 2. Materials and Methods

### 2.1. Microorganism 

*Synechocystis salina* strain LEGE 06155 (S. *salina*) was used for this study, and obtained from the Blue Biotechnology and Ecotoxicology Culture Collection (LEGE-CC)—CIIMAR (Centre of Marine and Environmental Research of University of Porto, Porto, Portugal). 

### 2.2. Cyanobacterial Cultivation Conditions

The growth of *S. salina* strain LEGE 06155 was performed in 2 L-round flasks (working volume of 1.8 L) in BG_11_ culture medium [27], supplemented with 10 g·L^−1^ of NaCl, with pH 8.9 ± 0.1 previously adjusted with CHES buffer (PanReach, AppliChem, Chicago, IL, USA), with temperature maintained at 23 °C. Agitation was provided via continuous air bubbling at the bottom of the cultures, using an airflow of 0.75 L_air_ ·L_culture_^−1^·min^−1^. Each run departed from an optical density (OD) of 0.1 (OD = λ_680_ nm − λ_750_ nm), which was kept in batch over 22 days, using the same conditions as for the pre-inoculum. The pre-inocula were maintained under a light intensity of 150 µmol_photons_·m^−2^·s^−1^, with light provided by a white LED (GreenPowerReasearch module white, 15 W, Phillips, Amsterdam, The Netherlands) using a light/dark cycle (L:D) of 16 h:8 h. 

### 2.3. Experimental Design 

**Figure 1:**
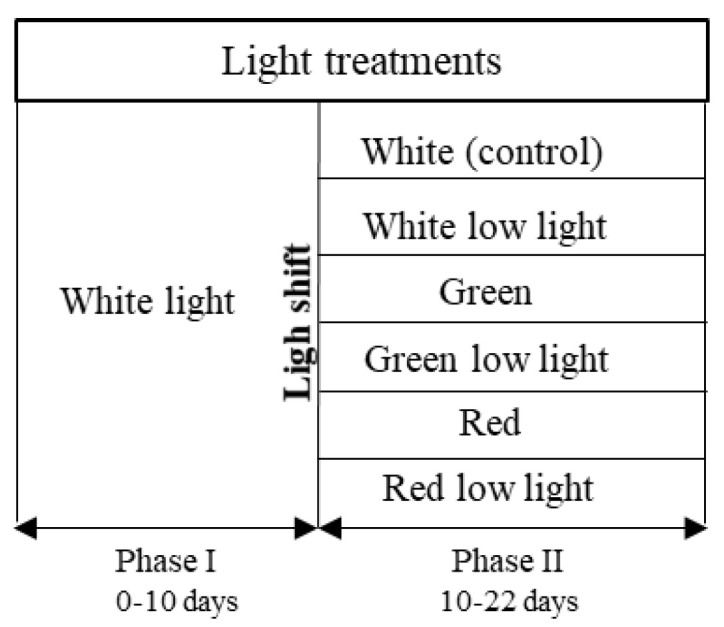
Scheme of experimental design regarding light treatments applied to *S. salina* LEGE 06155 cultures: white (control, 150 μmol_photons_·m^–2^·s^–1^), white low light (50 μmol_photons_·m^–2^·s^–1^), green (150 μmol_photons_·m^–2^·s^–1^), green low light (50 μmol_photons_·m^–2^·s^–1^), red (150 μmol_photons_·m^–2^·s^–1^), and red low light (50 μmol_photons_·m^–2^·s^–1^).

**Figure 2:**
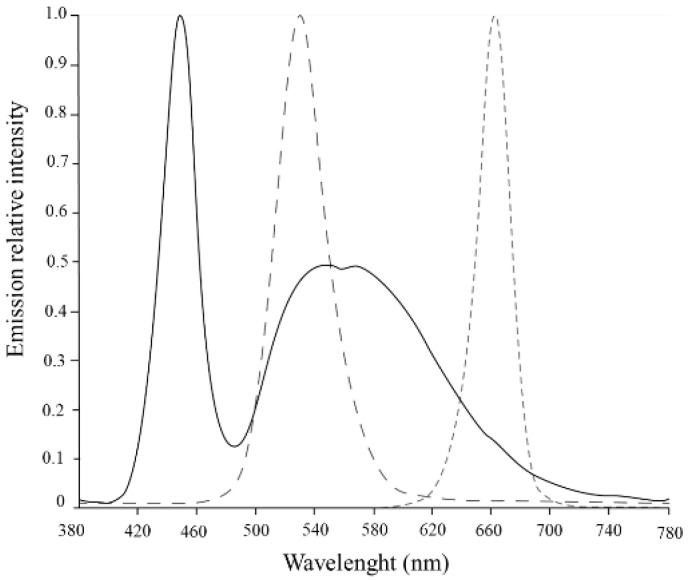
Emission relative intensity of LED spectra used in this study: white (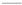); green (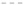) and red (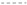).

*S. salina* was subjected to a two-phase light treatment over 22 days. In the first phase, cultures were subjected to white (W) light, containing energy from different wavelengths (λ_max_ = 450 nm, 540, and 580 nm), over 10 days for biomass growth. In the second phase, a light shock was imposed, with red (R) light (λ_max_ = 660 nm) and green (G) light (λ_max_ = 530 nm), both tested under 50 and 150 µmol_photons_·m^−2^·s^−1^ for light intensities, over 12 days. The cultures were also tested under low light conditions for white light, and a complete curve with white light under 150 µmol_photons_·m^−2^·s^−1^ was produced as control (Figure 1). Both green and red light were chosen for light shifts, since they have been reported to significantly influence PBP restructuration during cyanobacterial cell acclimatization and maintenance of photosynthetic efficiency [28,29]. The different light sources were placed below the cultures; the white LED (GreenPowerReasearch module deep red, 15 W, Phillips, The Netherlands) and red LED (GreenPowerReasearch module deep red, 10 W, Phillips, The Netherlands) consisted in a rack with 3 stripes of built-in lights, and the green LED (Systion, Portugal) consisted in a rack with 5 LED tubes. The light intensities were checked using a universal light meter with PAR sensor (UML-500 Universal, Walz, Effeltrich, Germany). Relative emission spectra of each LED (Figure 2) were measured with a spectroradiometer Lighting Passport Pro (model ALP-01, AsenseTek, Gatineau, QC, Canada), and the corresponding data were collected with software Spectrum Genius (AsenseTek, Canada, 2021).

### 2.4. Determination of Cyanobacterial Biomass Growth and Production

The cyanobacterial growth and biomass production were determined via dry weight (DW), with samples collected at defined intervals—namely by 0, 1, 4, 6, 8, 10, 11, 15, 18, 20, and 22 days. 

The DW was obtained by filtering aliquots of 2 mL of culture through preconditioned 0.45 µm (pore size) glass microfiber filter paper (Whatman GF/C, Maidstone, UK), and further dried at 100 °C until constant weight. Three biological replicates were performed, and DW analysis was performed in duplicate. 

The maximum specific growth rate (µ_max_, d^−1^) was determined by numerical regression to experimental data. The maximum biomass productivity (P_x_, mg L^−1^ d^−1^) was determined using DW experimental data based on the following equation:

Px (t) = (Xt − X0)/(t − t0) (1)

where X_0_ denotes biomass at the beginning of the experiment (time t_0_) and X_max_ represents maximum biomass concentration achieved at time t_._ The pH was also regularly monitored with a pH meter (Hanna Instruments, model HI2210). 

### 2.5. In Vivo Chlorophyll a Fluorescence Monitoring

Photosynthetic activity was evaluated by in vivo chlorophyll *a* fluorescence monitoring with pulse modulation (PAM), using a Junior-PAM/White fluorometer (Walz, Germany) [30,31]. Rapid light curves (RLC) were performed according to Figueroa et al. (2014) [32], in triplicate, with filtered circles of *S. salina* in contact with the fiber (90-degree angle) fixed at the center of the circle. RLC offers information about the overall photosynthetic efficiency state, as well as such other features as electron transport rates (i.e., saturation) of a photosynthetic organism. Maximal quantum yield of PSII—photosystem II-(Fv/Fm), maximum electron transport rates (ETR), and maximum non-photochemical quenching (NPQ) were duly calculated. Before measurement, samples were prepared by diluting a small volume of fresh culture with distilled water to an OD = 0.1, up to a final volume of 1 mL. This sample was filtered through a 0.45 nm filter paper Whatman (pore size 0.45 nm), and the biomass was then incubated in darkness for 15 min to assure that all photosynthetic reaction centers were closed. The samples were subjected to increased light pulses, from a basal state to a saturating pulse (up to 1500 µmol_photons_·m^−2^·s^−1^) to estimate Fv/Fm [30,31]. 

The ETR_max_ was calculated based on ETR = Y(II) × EPAR × fAQPSII, where Y(II) is the effective quantum yield, EPAR is the photosynthetic active radiation (PAR), A is the absorbance such that A = 1–10^−ODPAR^, with ODPAR being the OD used to measure the chlorophyll *a* fluorescence in the PAR region (0.1); finally, fAQPSII is the fraction of absorbed quanta to PSII in the PAR region of the spectrum, with a typical value of 0.36 for cyanobacteria [33]. Non-photochemical quenching (NPQ) was calculated using (Fm − Fm′)/Fm′, where Fm is the maximal fluorescence following application of saturation light pulse after 15 min incubation in darkness, and Fm′ is the maximal fluorescence upon application of saturation light pulse after 20 s incubation at each light intensity [34]. The RLC was fitted to a mathematical function to find the maximal ETR (ETR), expressed in μmol_electrons_·m^−2^·s^−1^, and the maximum non-photochemical quenching (NPQ).

### 2.6. Pigment Extraction and Quantification

#### 2.6.1. Pigment Extract Preparation

Extraction of chl *a*, total carotenoid, and PBPs were performed with ethanol and water, respectively, in sequential extraction (at 0, 1, 4, 6, 8, 10, 11, 15, 18, 20, and 22 days). To analyze carotenoid total content, aliquotes of 3 mL of fresh culture were collected and centrifuged (2744 g × 10 min); the supernatant was discarded, and 3 mL of ethanol was added to the remaining pellet with glass beads. Extraction was performed via bead beater in a *Precellys* homogenizer (Bertin Technologies, Montigny-le-Bretonneux, France), using a 6 min cycle at 8000 rpm (30 s homogenization with 40 s pause). The extracts were centrifuged (2744 g × 10 min), and the supernatant was kept under dimmed light and transferred to 5 ml Eppendorf’s for storage at −4 °C until pigment content determination. In extraction of PBPs, 3 mL of distilled water was added to the remaining pellet, and then vortex stirred over 20 s. Extracts were centrifuged at 2000 *g* over 8 min, and kept in dark for further content determination.

#### 2.6.2. Determination of Pigment Content 

The chl *a* and total carotenoid content were assessed via spectrophotometry (Shimadzu UV-1800, USA), according to Lichtenthaler and Buschmann (2001) [35]. Absorbance was read at λ = 470, λ = 652 and λ = 665 nm, and the content calculated as follows:

Chl *a* (μg·mL^−1^) = (16.72 A_664_) − (9.16 A_648_) (2)

Total carotenoids (μg·mL^−1^) = (1000 A_470_) − (1.63 Chl *a*) / 209 (3)

Results were expressed as concentration (mg·L^−1^) and maximum productivity (mg_car·_L_culture_^−1·^d^−1^). 

For PBPs quantification, namely phycocyanin (PC), allophycocyanin (APC), and phycoerythrin (PE), the aqueous extracts were spectrophotometrically read at λ = 562, 615 and 652 nm; Bennett and Bogorad’s equations [36] were then applied as follows:

PC (mg·mL^−1^) = (A_615_) − 0.474 A_652_)/5.34 (4)

APC (mg·mL^−1^) = [(A_652_) − (0.208 A_615_)]/5.09 (5)

PE (mg·mL^−1^) = [(A_562_) − 2.41(PC) − 0.849 (APC)]/9.62 (6)

The PBP content was expressed as concentration (mg·L^−1^) and in terms of maximum productivity (mg·L^−1^·d^−1^). 

### 2.7. Carotenoid Profile and Quantification

To ascertain whether there was a change in carotenoid profile between before and after the light treatments, a more detailed analysis was performed on the 10th day (before the light shock treatment) and on the last day (22nd day) of culture, for the sake of comparison. The samples were prepared by collecting 10 mL of fresh culture, and then centrifuged (2744× *g* × 10 min). The supernatant was discarded, and 5 mL of pure acetone was added to the remaining pellet, as well as 100 µL of internal standard solution of *trans*-β-Apo-8′-carotenal (170 mg·L^−1^; Sigma-Aldrich ≥ 96.0% (UV)]. Glass beads were also added to the samples for further *Precellys*-mediated homogenization (8000 rpm, 6 min cycle over 40 s homogenization, 30 s pause). The extracts were centrifuged (2744 g × 10 min) and kept at −20 °C until further analysis. The identification and quantification of carotenoids by HPLC was performed by a method described elsewhere [37]. The extracts were accordingly evaporated in a vacuum rotary evaporator, and resuspended in 400 µL of acetone:ethyl acetate (9:1) following previous filtration with a PTFE filter syringe (Membrane solutions, 0.22 µm) before injection. The HPLC used (Waters Alliance 2695, Milford, MA, USA) was equipped with LichoCart 250–4 C18 reverse-phase column (250 × 4 mm, 5-μm bondapack) (Merck, Darmstadt, Germany) (stationary phase), an FLR detector (Waters, Milford, MA, USA), a photodiode array (PDA) (Waters, USA) set to spectrum scanning within the range of λ = 250–750 nm, and a column heater (Waters, USA) to maintain a constant temperature throughout the analysis. 

Ethyl acetate (solvent A) and acetonitrile:water 9:1 (*v*/*v*) served as mobile phase (solvent B); a gradient with these solvents was established over time as follows: 0–31 min (0–60% A); 31–36 min (60% A); 36–38 min (60–100% A); 38–43 min (100% A); 43–50 min (100-0% A); and 50–55 min (0% A). During the elution process, the column was maintained at 25 ± 2 °C under a steady pressure of 3000 bar, with an overall flow rate of 1 mL·min^−1^. Spectrum data from all peaks were collected within the range 250–750 nm. 

The pigments were identified by the extracts UV–Vis spectra, and by comparison of retention times (RT) with standards of zeaxanthin (Extrasynthese, ≥98.0% (UV) with RT = 13.2 min, chl *a* (Sigma, ≥96.0% (UV)) with RT = 24.6 min, echinenone with RT = 25.2 min, and β-carotene (Extrasynthese, ≥98.0% (UV)) with RT = 32.8 min; and quantified via calibration curves encompassing said standards. Three biological replicates were analyzed, and results were expressed as µg·g_Dw_^−1^. Total carotenoid productivities were expressed as mg_car_·L^−1^·d^−1^. 

### 2.8. Statistical Analysis

The experimental data were analyzed using software GraphPad Prism, version 8 for Windows (GraphPad Software, San Diego, CA, USA). Data analyzed were submitted to Shapiro–Wilk test, to confirm the normal distribution of residuals. Then, a one-way ANOVA with Tukey’s multiple comparison tests was used to assess variance between total content of chl *a*, total carotenoids, and PBPs.

## 3. Results 

### 3.1. Growth and Biomass Production

**Figure 3:**
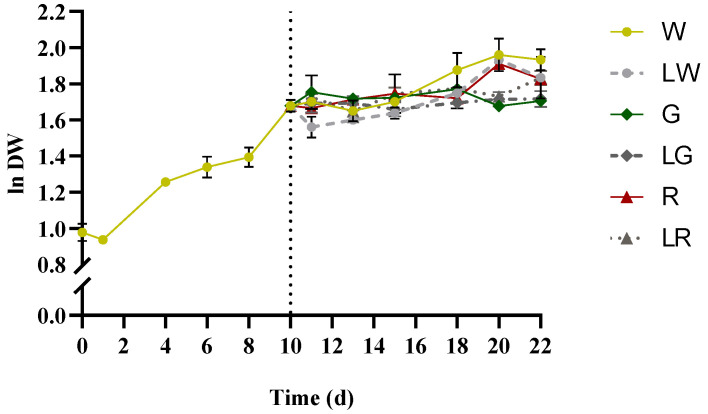
Growth curves of *S. salina* LEGE 06155, expressed as logarithm of dry weight (ln DW) versus time (per day). The vertical dotted line denotes the separation between phase I: treatment of W (white, 150 μmol_photons_·m^−2^·s^−1^) (control), from 0 to 10th days (left) and phase II: LW (low white, 50 μmol_photons_·m^−2^·s^−1^), G (green, 150 μmol_photons_·m^−2^·s^−1^), LW (low green, 50 μmol_photons_·m^−2^·s^−1^), R (red, 150 μmol_photons_·m^−2^·s^−1^), and LR (low red, 50 μmol_photons_·m^−2^·s^−1^) from 10th to 22nd days (right). The data points show average ± SD, n = 3.

*S. salina* was found to grow under all light treatments applied, after 10 days with white light treatment. At early growth, some lag phase became apparent, which rapidly entered the exponential phase between 1–4 days, with μ_max_ = 0.18 ± 0.00 d^−1^ (Figure 3). The curve of the white treatment presented an average productivity of 0.18 ± 0.01 mg·L^−1^·d^−1^ within 0–22 days. No significant growth patterns regarding green and low green light treatments were recorded (*p* > 0.05); yet white light, low white, red, and red under low light intensity seemed to affect positively the biomass, with μ_average_ = 0.17 ± 0.01 d^−1^. Biomass productivities were hereby considered as the average productivities, taking into account the period 0–22 d; no significant differences were recorded among them (*p* > 0.05), with an average value of P_x_ = 0.17 ± 0.01 g·L^−1^·d^−1^, except for green and low green treatments that presented (a lower) P_x_ = 0.13 ± 0.00 g·L^−1^·d^−1^ (Table 1). 

### 3.2. Photosynthetic Efficiency

**Figure 4:**
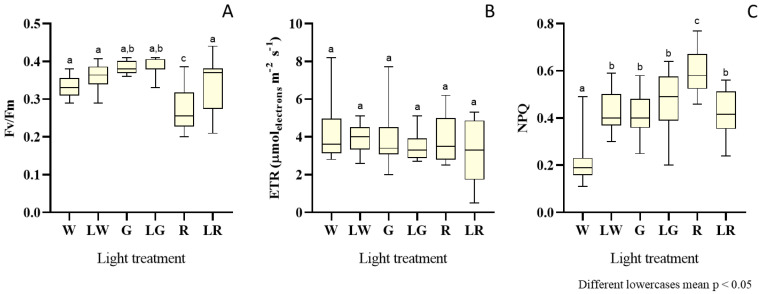
Box and whisker plots of the data of photosynthetic efficiency, for each light treatment: W (white, 150 μmol_photons_·m^−2^·s^−1^) (control), LW (low white, 50 μmol_photons_·m^−2^·s^–1^), G (green, 150 μmol_photons_·m^−2^·s^−1^), LW (low green, 50 μmol_photons_·m^−2^·s^−1^), R (red, 150 μmol_photons_·m^–2^·s^–1^), and LR (low red, 50 μmol_photons_·m^−2^·s^−1^) applied to *S. salina* LEGE 06155, considering Fv/Fm (maximum quantum yield in PSII) (**A**), ETR (electron transport rate) expressed in μmol_electrons_·m^−2^·s^−1^ (**B**), and NPQ (non-photochemical quenching) (**C**). The horizontal line within the box indicates the median, boundaries of the box indicate the 25th and 75th quartile, and whiskers indicate the lowest and the highest values of the results. The lowercase letters denote statistically significant differences (*p* < 0.05).

*S. salina* was evaluated regarding its photosynthetic efficiency, in terms of Fv/Fm, ETR_max_, and NPQ (Figure 4). Results were presented as box and whiskers plots (Figure 4), owing to the high variance in data points. Figure 4A depicts the distribution of results regarding Fv/Fm for each light treatment. It unfolded significantly lower trends of Fv/Fm values (i.e., median = 0.26), for *S. salina* grown under red light (high intensity), than in other treatments (*p* < 0.05). A higher distribution trend was observed for the white and green treatment (*p* < 0.05), in the order of 0.36. 

Regarding ETR or electron transport rate (Figure 4B) for the photosynthetic apparatus in the PSII (or rate at which electrons move through the photosynthetic chain), the trends are quite similar for all treatments–except for the white treatment under low intensities, which exhibited a slightly higher median (median = 4 μmol_electrons_·m^−2^·s^−1^); however, this does not materialize a significant difference relative to the other treatments (*p* > 0.05). 

The results in terms of NPQ, or non-photochemical quenching, are presented in Figure 4C. The lowest NPQ values (median = 0.19) were found for *S. salina* treated with white light under high intensity. The highest value for this parameter was observed for the treatment with high red light (median = 0.58). NPQ for the other light treatments yielded similar median values (i.e., 0.43), without significant differences between them (*p* > 0.05).

### 3.3. Effect of Light Treatment on Pigment Content and Profile

#### 3.3.1. General Pigment Content

**Figure 5:**
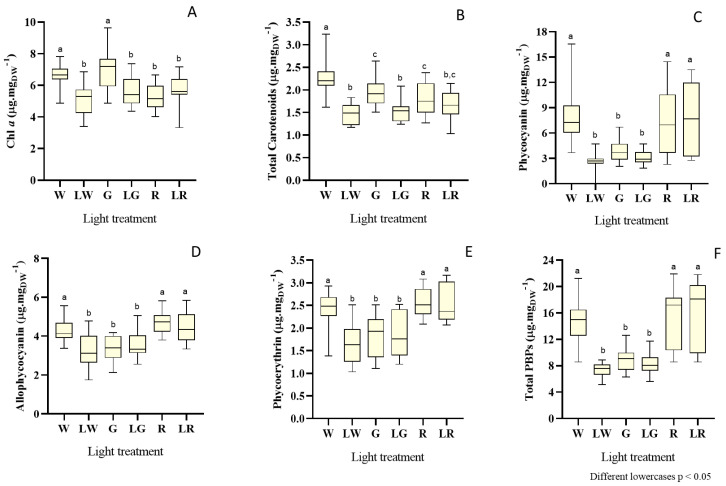
Box and whisker plots of pigment content, expressed in mg g_DW_^−1^, for each light treatment: W (white, 150 μmol_photons_·m^−2^·s^−1^) (control), LW (low white, 50 μmol_photons_·m^−2^·s^−1^), G (green, 150 μmol_photons_·m^−2^·s^−1^), LW (low green, 50 μmol_photons_·m^−2^·s^−1^), R (red, 150 μmol_photons_.m^−2^.s^−1^), and LR (low red, 50 μmol_photons_·m^−2^·s^−1^) applied to *S. salina* LEGE 06155, considering chl *a* (**A**), total carotenoids (**B**), phycocyanin (**C**), allophycocyanin (**D**), phycoerythrin (**E**), and total phycobiliproteins (**F**). The horizontal line within the box indicates the median, the boundaries of the box indicate the 25th and 75th quartiles, and the whiskers indicate the lowest and the highest values of the results. The lowercase letters denote statistically significant differences (*p* < 0.05).

As the fluctuations of general pigment content over time were quite pronounced, the results are presented in the form of box and whiskers plots (Figure 5). In general, distinct light wavelengths and combined light intensities significantly impacted the content of different pigments, including chl *a*, total carotenoids, and phycobiliproteins. 

Considering the content of chl *a*, one of the most important pigments in photosynthesis, the highest values were observed for treatment under green light, at high intensities (median = 7.31 mg·g_DW_^−1^, day 13). However, chl *a* presented lower content trends when red light treatments (both under low and high intensities) and green light treatment (with low intensity) were applied (Figure 5A) (median = 5.32 mg·g_DW_^−1^).

Results on total carotenoids showed that white, green, and red treatments, under higher intensities, tend to influence the accumulation of these pigments to higher concentrations, when compared with the same treatments under low light intensities (Figure 5B). The highest concentration recorded was under white light treatment for high intensity, with a median of 2.18 mg·g_DW_^−1^ and a major peak of 3.23 mg·g_DW_^−1^ at day 15. 

Concerning phycobiliproteins content, the first remark should focus on the order of magnitude of the values for the content of each PBP individually—at least for the treatment with white light (under high intensities) and red light (both under low and high intensities). PC produced high peaks, with values ranging in 13.59–16.54 mg·g_DW_^−1^, with the best value obtained for the white treatment under high intensity on day 16 (Figure 5C); APC took values between 4.18–5.84 mg·g_DW_^−1^ (the maximum under red treatment for low intensity) (Figure 5D), and PE reached a maximum of 3.17 mg·g_DW_^−1^ (in the red treatment under low intensity) on day 20 (Figure 5E). For total PBP, both red treatments (under low and high intensities) impacted positively the content of PBPs, without significant differences between them (*p* > 0.05), and a maximum value of 21.89 mg·g_DW_^−1^ on day 22. The difference between treatments with green light (irrespective of light intensity) and also white treatment (low intensity) is apparent, as the latter followed a negative trend of accumulation of the whole of these pigments. 

#### 3.3.2. Carotenoid Profile

The carotenoid profile was assessed before application of each light treatment during cultivation (10th day), and on the last day of cultivation (22nd day) to assess differences across all light treatments (Table 2). Overall, the carotenoid profile did not change across all light treatments, but the content did generally increase. The highest differences were observed for white treatment (under high intensity), in particular for zeaxanthin and β-carotene (*p* < 0.05). The green treatment also stood out, doubling the content of zeaxanthin, echinenone, and β-carotene. The content in lycopene was outstanding, with almost 2.6-fold following application of the red treatment. 

### 3.4. PC/PE Ratio

As light influences directly the ratio between PC and PE in cyanobacteria, as a response to acclimatization under different light conditions, the ratio between these two pigments was analyzed right before application of the light shifts (phase I, 10th day), during treatment (phase II, 15th day) and by the end of light treatment (phase II, 22nd day) (Table 3). An increasing trend was observed with cultivation time for all treatments, except the low green treatment. The most prominent increase was found under the low red treatment, in particular from the 10th day on, and for both days 15 and 20. On the other hand, the lowest increase was recorded under low green treatment, with a significant increase in phycoerythrin by the 22nd day, when compared to both 10th and 15th day (*p* < 0.05). 

###  3.5. Pigment Productivities

Pigment productivities were assessed for all treatments applied to *S. salina*; maximum peaks of productivity varied to a significant extent (*p* < 0.05) (Table 4). In addition, those peaks were not always recorded on the same days for some treatments. The highest productivity of chl *a* was obtained for white treatment (control), on the 8th day of cultivation. On the other hand, productivities for total carotenoids were achieved at later stages (i.e., stationary phase) of cultivation, with the highest value obtained for white treatment, 0.32 ± 0.01 mg·g^−1^·L^−1^ (i.e., more than double when compared to the same treatment under low intensities). For PC, both red and white treatments (high intensity) were ≈17-fold the lowest productivity achieved, under white treatment and low intensity. However, the highest productivity of PC took 22 days under white light treatment, whereas the second highest value (under low red treatment) attained the highest productivities earlier, i.e., on the 15th day. Both APC and PE exhibited higher productivities for white light treatment (high intensity), but for distinct periods of time (6th and 22nd days, respectively). For total PBPs, white and red treatments tend to increase the production of these pigments without significant differences (*p* > 0.05). The maximum productivity achieved for red treatment (high intensity) (15th day) was almost 7-fold that of the lowest value achieved for white treatment under low intensity. 

## 4. Discussion

Light quality and intensity play an important role in cyanobacterial biomass and photosynthetic pigment production [15]. In this study, *S. salina* was subjected to different treatments with LEDs (white, green, and red, combined with two different intensities) in two distinct phases. The effect of light modulation upon bioactive pigment productivities, and how this cyanobacterium responds to different light treatments applied were evaluated. 

### 4.1. Biomass Production and Photosynthetic Efficiency

Regarding growth and biomass production (and apart from the control), emission spectra of red LED (irrespective of light intensity) were shown to positively impact biomass production by *S. salina*. Several studies have demonstrated red light to be an external stimulator of increased growth in some species of cyanobacteria, (e.g., *Arthrospira platensis, Cyanobium* sp.), possibly correlated with a peak absorption of chl *a* and PBP increased production [38,39,40,41,42]. However, chl *a* content did not stand out for red light treatment, and no linear correlation between biomass concentration and this pigment was observed (except for the white treatment under high intensity, data not shown). Similar results were previously reported by Mohsenpour et al. (2012) [43] for microalga *Chlorella vulgaris* and cyanobacterium *Gloethece membranacea* subjected to violet, green, orange, or red light treatments. For cyanobacteria, due to presence of PBPs, this relationship cannot be so straightforwardly established. The red treatment leads indeed to a higher content in total PBPs, which could relate to the complementary chromatic adaptation or CCA–according to which the ratio of PC and PE is altered, and can produce a more efficient photosynthetic process [6]. Results for photosynthetic efficiency were, nevertheless, inconsistent with these data—at least for the red treatment under high light intensity. Fv/Fm considers the ratio between variable and maximum fluorescence of chl *a*, which indicates the maximum quantum yield of PSII—and an indicator of “good health” of the photosynthetic apparatus in the organism at stake; the higher its value, the best their maximum quantum yield, and probably the higher the growth and biomass productivities. This value was high for all treatments, except red light under high intensity, thus indicating poor photosynthetic performance–probably induced by saturation of the photosystem. Additionally, NPQ values exhibited an elevated value that unfolds a loss of efficiency in the photosynthetic apparatus, meaning that energy is not harnessed for photochemical reactions, but probably turns out dissipated as heat [30]. NPQ is a photoprotective mechanism present in cyanobacteria, but also in other photosynthetic organisms (e.g., plants, algae), which relies on the conversion and dissipation of the excess excitation energy to heat. In cyanobacteria, this mechanism involves conformational changes of an orange carotenoid protein (OCP) that binds to the phycobilisome and leads to energy quenching in the phycobiliproteins–thus significantly decreasing the amount of energy reaching the PSII reaction centers [44]. This mechanism is usually activated under blue-green light; however, OCP was demonstrated to play an important role in photoprotection under strong orange-red light, by decreasing the singlet oxygen concentration, potentially causing oxidative stress of the cells [44,45]. Possibly under the red treatment, the dissipation of heat by NPQ is promoted by other routes, including carotenoids (e.g., β-carotene, zeaxanthin) present near the highly excited chl *a* at the core of the photosynthetic apparatus. Moreover, the ETR did not show significant alterations, as the electron transfer rate can be maintained if this extra energy is freed through the NPQ mechanism; hence, the photosynthetic system is probably saturated [46]. 

On the other hand, the white control was shown to be the best for biomass growth and productivity. This is consistent with data regarding the highest chl *a* and total PBPs content, concomitant with the pertaining to photosynthetic efficiency with increased Fv/Fm, increasing trend of ETR, and low NPQ. The efficient photosynthetic performance relates to the absorption spectra of pigments overlapping LED wavelengths (i.e., 450 and 540 nm). Therefore, PBPs efficiently funnel the energy to chl *a* that is directly transferred to the PSII reaction centers, with minimal losses in energy efficiency (i.e., reduced NPQ). 

Significant differences in kinetic parameters, in terms of light intensities, were not found. The photosynthetic rate is directly proportional to light irradiance; light intensity below light saturation point leads to increased photosynthetic performance, along with higher biomass productivities and rates of CO_2_ uptake [47]. If light overcomes the critical point of light saturation, then cultures enter a state of photooxidation– which potentially leads to damage in the photosystem apparatus and would possibly compromise overall photosynthetic efficiency, thus jeopardizing the growth of those microorganisms. For this study, it seems that the light intensities chosen did not reach the level that is common for light saturation, as photoinhibition was observed in none of those light treatments. This observation is also corroborated by the content of total carotenoids and chl *a*. Cyanobacterial accumulation of chl *a* and carotenoids constitutes an important factor in the regulation of photosynthesis and growth [48,49]. In general, under high light intensities—and usually when the saturation point is overpassed—chl *a* tends to substantially decline, (i.e. Car/Chl *a* increases) due to photoinhibition [50]. In this work, both chl *a* and carotenoids underwent a positive response under all tested wavelengths at high light intensities (150 μ_photons_·m^−2^·s^−1^); it seems that light curves did not attain a plateau level or photoinhibition, and that both chl *a* and carotenoids accumulation are favored, as cells tend to optimize the capture of available light. 

### 4.2. Acclimatization and Pigment Content and Profile

In this study, all pigments evaluated were influenced by the quality (and intensity) of light; and *S. salina* was able to respond to the environmental alteration by readapting its photosynthetic pigment antenna. On the one hand, green light (high intensity) favored accumulation of chl *a*, whilst total carotenoids were produced under white light (control); on the other hand, white treatment (control) and red light (both intensities) stimulated production of both PBPs, and each one individually. The accumulation of chl *a* under green light was also previously observed for cyanobacterium *Gloeothece membranacea*, which showed an increase of 1.12- and 1.6-fold when compared to the control (white light) [43]. *Cyanobium* sp. subjected to five different light conditions (white, green, red, blue, and UV) also preferred green light for chl *a* accumulation [40]. In fact, green light has been reported to be the light less efficiently absorbed [51]; however, its supplementation can help deepen penetration into dense cyanobacterial cultures, and become more absorbed than other wavelengths [52]. 

Total carotenoids were favored by the high light intensity in the white treatment; the effects of green treatment and red light under high intensities (in addition to white treatment) upon total carotenoid content are also noteworthy, including some carotenoids individually: results of lycopene (red), zeaxanthin (green), echinenone (green), and β-carotene (green) can be explained as part of the strategy for acclimatization. In cyanobacteria, carotenoids have the main role in energy dissipation and protection against oxidative damage [15,53]. Some studies show that, depending on the species, some cyanobacteria are more impacted by green or red light in terms of enhanced carotenoid production. For instance, *Pseudanabaena* sp. increased its content of carotenoids by 30%, under green light when compared to white light [54]; this contradicts the results for *Cyanobium* sp. with two-phase cultivation, using white and red LEDs–with a 50% increase in carotenoids under the red phase when compared to the white phase [21]. These differences can be related not only to the PBP composition of each species, but also to the possible existence of a green-red photoreceptor involved in carotenoid production by certain cyanobacteria [53].

The total PBP production and each PBP individually were chiefly promoted by red light treatment (apart from control, *p* > 0.05). These pigments tend to absorb under a yellow-red range, and several studies point at the red light as a stimulator of biomass growth and increased production of PBPs (in particular PC) [11,40,55,56]. For instance, in *Arthrospira platensis* subjected to different light qualities using LED (red, white, green, and yellow), PC was preferentially stimulated under the red spectrum [55]. These pigments have massive importance for the mechanisms of adaptation and regulation to different types of light; most cyanobacteria possess CCA mechanisms that help in transient reconfiguration of their phycobilisome, with an alteration of PC/PE ratios, in order to better adapt the organism to the quality and quantity of incident radiation from the environment [6]. In addition, green/red light modulation produces a response in PC/PE levels, and an alteration in the corresponding absorption properties of the phycobilisome [6,57]. *S. salina* seems to fit in Type III acclimatization. The application of light shock supported increase in the PC/PE ratios under red treatments (particularly at low intensity); whereas the green treatment led to a slight increase in PE over time. PE absorbs under the green range, and this realization agrees with said results. Moreover, literature reports have it that PBP production is preferentially obtained under low to medium intensities of light, since high light intensities can cause photoinhibition [12]. However, the results are somehow contradictory, because the effect of light intensity was not relevant across the red treatments—it instead stood out for white light under high intensity. Possibly for both white and red lights, *S. salina* can stand higher intensities as the maximum photoinhibition threshold has not yet been reached. Recall that the overall process of chromatic adaptation is not straightforward, and is highly strain-dependent. In addition, it depends on the physiological age of the cultures, and the genetic/molecular regulation of the chromatic adaptation itself [6,57]. 

### 4.3. Pigment Productivities and Biotechnological Perspectives 

In light of a biotechnological perspective, chromatic light effects could be used as a tool to enhance production of certain natural pigments in cyanobacterial cultures. Therefore, this study provided a few hints about the strategy to follow with LED treatment/modulation for this species of cyanobacterium, depending on the target pigment. The use of LEDs should also meet requirements in terms of growth and biomass accumulation; in this work, both specific growth rate and biomass productivities seemed quite low when compared to the literature, even under the first stage of cultivation used for control. For instance, *S. salina* is able to achieve maximum growth rates of 0.3 d^−1^ under fluorescent light conditions (100 μmol_photons_·m^−2^·s^−1^), with productivities of ca. 1.5 g·L^−1^·d^−1^ (data not shown). LED quality does not seem to be limiting to *S. salina* growth, but the chosen intensities were apparently low. In fact, it has been reported that *Synechocystis* (strain PCC 6803) can stand up to 500 μmol_photons_·m^−2^·s^−1^ with high growth and photosynthetic performance (under a specific temperature of 30 °C and pH 8); however, photoinhibition occurs when light intensity exceeds 800 μmol_photons_·m^−2^·s^−1^ [38]. Therefore, we believe that higher growth rates, biomass production, and possibly enhanced pigment production could be obtained by increasing the light intensity of LED; unfortunately, the selected intensities could not be further increased, due to LED equipment limitations. 

In terms of pigments, the exposure of *S. salina* to green light (high intensity) positively influenced the production of chl *a*, whilst white light enhanced carotenoid productivities in addition to positively influencing production of zeaxanthin and β-carotene; these are important pigments for use by the food and aquaculture industries as nutraceuticals or colorants [15,53]. Focusing on PC, one of the most important pigments produced by *S. salina* and important for their market value (noting that these pigments are expanding in the market, due to their unique colorant and antioxidant properties [5]), red shock treatment could be chosen to enhance their productivities. This selection seems more suitable than white light, because the latter takes 5 more days to reach a similar productivity. A study by Pagels et al. (2020) [21] also unfolded the positive effect of the two-phase, white-red light treatment strategy; PC productivities were improved within 14 days (10 days of white + 4 days of red), instead of needing 21 days of cultivation. 

Our option for low intensity may also be regarded as a cost-saving one. The energy consumption by the process is a relevant parameter that affects the whole microalgal production costs. In view of this, low energy consumption techniques might be preferred over high energy consumption regarding artificial light [58]. In addition, the use of LED-tailored lighting will probably be elected in the near future as the light source for microalgal and cyanobacterial biomass cultivation at large scale, and concomitant production of specific pigments and other value-added compounds (at least indoors). They entail indeed lower energy consumption, lower maintenance requirements, and wider lifespan than common fluorescent light. A few studies conducted with light modulation for cyanobacteria have emerged in recent years [20,21,55,59,60], as LED quality and efficiency have been steadily increasing [20].

Finally, fundamental studies pertaining to light modulation are quite important to pinpoint and understand which optimization strategies are to be followed toward optimization of the content of desired pigments. This study, in particular, showed the capacity of *S. salina* to acclimate to different light conditions, fitting in type III of acclimatization. From a biotechnological perspective, the modulation with white and red light (10 days of white + 5 days of red) proved a relevant strategy to enhance PC productivity. There is no need to cultivate this cyanobacterium over 22 days, because similar productivities can be achieved 7 days earlier when compared to white light. In an commercial microalgal bioprocess, this strategy may have significant impacts regarding financial and energy savings, a deed quite appealing from a biotechnological perspective. 

## Figures and Tables

**Table 1:** Kinetic parameters determined for growth of phase II of *S. salina* LEGE 06155 cultivation: µ_average_ (average specific growth rate)_,_ expressed in d^−1^, and P_x_ (average productivity), expressed in g·L^−1^·d^−1^ for each applied light treatment: W (white, 150 μmol_photons_·m^−2^·s^−1^) (control), LW (low white, 50 μmol_photons_·m^−2^·s^−1^), G (green, 150 μmol_photons_·m^−2^·s^−1^), LW (low green, 50 μmol_photons_·m^−2^·s^−1^), R (red, 150 μmol_photons_·m^−2^·s^−1^), and LR (low red, 50 μmol_photons_·m^−2^·s^−1^). The lowercase letters denote statistically significant differences across columns (*p* < 0.05).

Light Treatments	µ_average_ (d^−1^)	P_x_ (g·L^−1^·d^−1^)
W (control)	0.015 ± 0.00 ^a^	0.18 ± 0.00 ^a^
LW	0.014 ± 0.01 ^a^	0.17 ± 0.00 ^a^
G	0.000 ± 0.00 ^b^	0.13 ± 0.00 ^b^
LG	0.000 ± 0.00 ^b^	0.13 ± 0.00 ^b^
R	0.012 ± 0.00 ^a^	0.16 ± 0.00 ^a^
LR	0.011 ± 0.00 ^a,c^	0.15 ± 0.00 ^a,c^

**Table 2:** Carotenoid profile and content for light treatment applied on the 10th day of cultivation of *S. salina* LEGE 06155: W (T10) (white, 150 μmol_photons_·m^−2^·s^−1^) and for the 22nd day of cultivation, for the light treatments applied: W (white, 150 μmol_photons_·m^−2^·s^−1^), LW (low white, 50 μmol_photons_·m^−2·^s^−1^), G (green, 150 μmol_photons_·m^−2^·s^−1^), LW (low green, 50 μmol_photons_·m^−2^·s^−1^), R (red, 150 μmol_photons_·m^−2^·s^−1^), and LR (low red, 50 μmol_photons_·m^−2^·s^−1^). The results are reported as average ± SD (n = 3) and expressed in μg·g_DW_^−1^. For each row, different upper lowercase indicates *p* < 0.05. * Quantification was in trans-β-Apo-8′-carotenal (internal standard) equivalents.

Identified Pigments	Concentration of Pigment (µg·g_DW_^−1^)						
	W (T10)	W	LW	G	LG	R	LR
Lycopene *	0.86 ± 0.04 ^a^	2.19 ± 0.02 ^b^	0.82 ± 0.27 ^a^	1.33 ± 0.05 ^c^	0.89 ± 0.15 ^a,d^	2.30 ± 0.16 ^b^	1.02 ± 0.14 ^c^
Zeaxanthin	3.36 ± 0.24 ^a^	14.60 ± 0.52 ^b^	3.87 ± 0.65 ^a^	6.43 ± 0.17 ^c^	5.14 ± 0.06 ^c^	5.34 ± 0.54 ^c^	6.19 ± 0.22 ^c^
Echinenone	0.49 ± 0.02 ^a^	1.45 ± 0.18 ^b^	0.51 ± 0.01 ^a^	0.94 ± 0.01 ^c^	0.78 ± 0.03 ^d^	0.76 ± 0.04 ^d^	0.71 ± 0.07 ^d^
β-carotene	2.86 ± 0.02 ^a^	11.34 ± 0.70 ^b^	3.71 ± 0.37 ^a^	5.57 ± 0.44 ^c^	4.66 ± 0.22 ^c^	3.95 ± 0.17 ^a^	5.39 ± 0.46 ^c^
α-carotene	0.56 ± 0.03 ^a^	1.52 ± 0.14 ^b^	0.63 ± 0.16 ^a^	0.82 ± 0.05 ^c^	0.79 ± 0.04 ^c^	0.61 ± 0.01 ^a^	0.68 ± 0.02 ^a^

**Table 3:** Phycocyanin/phycoerythrin (PC/PE) ratio on 10th (phase I), 15th and 22nd days (phase II) of cultivation of *S. salina* LEGE 06155, for the light treatments: W (white, 150 μmol_photons_.m^−2^.s^−1^) (control), LW (low white, 50 μmol_photons_·m^−2^.·s^−1^), G (green, 150 μmol_photons_·m^−2^·s^−1^), LW (low green, 50 μmol_photons_·m^−2^·s^−1^), R (red, 150 μmol_photons_·m^−2^·s^−1^), and LR (low red, 50 μmol_photons_·m^−2^·s^−1^). The results are reported as average ± SD (n = 3) and expressed as %. For each row, different upper lowercase indicates *p* < 0.05.

		PC/PE Ratio (%)		
Light Treatment	Day 10 (Phase I)	Light Treatments	Day 15 (Phase II)	Day 22 (Phase II)
W (control)	60/40 ± 2.5 ^a^	W (control)	79/21 ± 3.2 ^b^	76/24 ± 2.6 ^b^
		LW	61/39 ± 3.9 ^a^	70/30 ± 3.2 ^b^
		G	69/31 ± 2.8 ^b^	68/32 ± 4.2 ^b^
		LG	64/36 ± 3.4 ^a^	44/56 ± 2.2 ^b^
		R	77/23 ± 1.1 ^b^	76/24 ± 3.1 ^b^
		LR	82/18 ± 2.0 ^b^	85/15 ± 0.5 ^b^

**Table 4:** Maximum productivities of pigments for light treatments applied to *S. salina* LEGE 06155: White (150 μmol_photons_·m^−2^·s^−1^) (control), low white (50 μmol_photons_·m^−2^·s^−1^), green (150 μmol_photons_·m^−2^·s^−1^), low green (50 μmol_photons_·m^−2^·s^−1^), red (150 μmol_photons_·m^−2^·s^−1^), and low red (50 μmol_photons_·m^−2^·s^−1^) applied. The lowercase letters denote statistically significant differences (*p* < 0.05) for each parameter analyzed (in column). Results are reported as average ± SD (n = 3) and expressed in mg·g^−1^·L^−1^.

	Maximum Pigment Productivities (mg·g^−1^·L^−1^)											
Light Treatment	Chl *a* (mg·g^−1^·L^−1^)	Day	Total Carotenoids (mg·g^−1^·L^−1^)	Day	Phycocyanin (mg·g^−1^·L^−1^)	Day	Allophycocyanin (mg·g^−1^·L^−1^)	Day	Phycoerythrin (mg·g^−1^·L^−1^)	Day	Total PBPs (mg·g^−1^ L^−1^)	Day
W (control)	1.03 ± 0.02 ^a^	6	0.32 ± 0.01 ^a^	15	1.42 ± 0.13 ^a^	22	0.33 ± 0.01 ^a^	6	0.27 ± 0.01 ^a^	22	1.52 ± 0.02 ^a^	22
LW	0.59 ± 0.03 ^b^	13	0.13 ± 0.0 ^b^	22	0.08 ± 0.00 ^b^	15	0.07 ± 0.01 ^b^	20	0.08 ± 0.01 ^b^	20	0.25 ± 0.04 ^b^	18
G	0.96 ± 0.00 ^c^	13	0.29 ± 0.00 ^c^	13	0.51 ± 0.02 ^c^	18	0.09 ± 0.00 ^b^	22	0.12 ± 0.01 ^c^	18	0.65 ± 0.03 ^c^	18
LG	0.72 ± 0.06 ^d^	11	0.13 ± 0.01 ^b^	22	0.19 ± 0.0 ^d^	18	0.11 ± 0.01 ^b,c^	20	0.11 ± 0.01 ^c^	15	0.40 ± 0.03 ^d^	18
R	0.59 ± 0.00 ^b^	15	0.19 ± 0.00 ^d^	15	1.29 ± 0.39 ^a^	15	0.21 ± 0.01 ^d^	15	0.27 ± 0.01 ^a^	15	1.70 ± 0.07 ^e^	15
LR	0.75 ± 0.00 ^d^	15	0.23 ± 0.02 ^e^	13	1.30 ± 0.04 ^a^	15	0.27 ± 0.02 ^e^	18	0.21 ± 0.00 ^d^	18	1.43 ± 0.03 ^a^	15

## Data Availability

The authors confirm that the data supporting the findings of this study are available within the article.
